# Photocatalytic and Electrocatalytic Properties of Cu-Loaded ZIF-67-Derivatized Bean Sprout-Like Co-TiO_2_/Ti Nanostructures

**DOI:** 10.3390/nano11081904

**Published:** 2021-07-24

**Authors:** Hye Ji Jang, So Jeong Park, Ju Hyun Yang, Sung-Min Hong, Choong Kyun Rhee, Youngku Sohn

**Affiliations:** 1 Department of Chemistry, Chungnam National University, Daejeon 34134, Korea; gpwldndud@naver.com (H.J.J.); jsjs5921@naver.com (S.J.P.); mil03076@naver.com (J.H.Y.); qwqe212@naver.com (S.-M.H.); ckrhee@cnu.ac.kr (C.K.R.); 2 Department of Chemical Engineering and Applied Chemistry, Chungnam National University, Daejeon 34134, Korea

**Keywords:** ZIF-67, Co-TiO_2_/Ti, photocatalytic CO_2_ reduction, electrocatalytic CO_2_ reduction, oxygen evolution reaction, water splitting

## Abstract

ZIF-derivatized catalysts have shown high potential in catalysis. Herein, bean sprout-like Co-TiO_2_/Ti nanostructures were first synthesized by thermal treatment at 800 °C under Ar-flow conditions using sacrificial ZIF-67 templated on Ti sheets. It was observed that ZIF-67 on Ti sheets started to thermally decompose at around 350 °C and was converted to the cubic phase Co_3_O_4_. The head of the bean sprout structure was observed to be Co_3_O_4_, while the stem showed a crystal structure of rutile TiO_2_ grown from the metallic Ti support. Cu sputter-deposited Co-TiO_2_/Ti nanostructures were also prepared for photocatalytic and electrocatalytic CO_2_ reduction performances, as well as electrochemical oxygen reaction (OER). Gas chromatography results after photocatalytic CO_2_ reduction showed that CH_3_OH, CO and CH_4_ were produced as major products with the highest MeOH selectivity of 64% and minor C_2_ compounds of C_2_H_2_, C_2_H_4_ and C_2_H_6_. For electrocatalytic CO_2_ reduction, CO, CH_4_ and C_2_H_4_ were meaningfully detected, but H_2_ was dominantly produced. The amounts were observed to be dependent on the Cu deposition amount. Electrochemical OER performances in 0.1 M KOH electrolyte exhibited onset overpotentials of 330–430 mV (vs. RHE) and Tafel slopes of 117–134 mV/dec that were dependent on Cu-loading thickness. The present unique results provide useful information for synthesis of bean sprout-like Co-TiO_2_/Ti hybrid nanostructures and their applications to CO_2_ reduction and electrochemical water splitting in energy and environmental fields.

## 1. Introduction

Metal–organic frameworks (MOFs) including other materials have been extensively employed in diverse application areas such as environments, adsorbents, organic light emitting displays, sensors and catalysts [1,2,3,4,5,6,7,8,9,10]. Among diverse MOFs, the zeolitic imidazolate framework (ZIF) exhibits zeolite-like topologies where imidazolate linkers are connected with tetrahedrally coordinated transition metal ions such as Co and Zn [1,2,3,4,5,6,7,8,9,10,11,12,13,14,15,16,17,18,19,20,21,22,23,24,25,26,27]. In this ZIF-class, ZIF-67 has Co-N tetrahedral coordination commonly synthesized with Co(II) precursor and 2-methylimidazole [5,6,7,8,9,10]. Many catalyst materials have been synthesized using ZIF-67 and examined in diverse areas of hydrogen evolution reaction (HER), oxygen evolution reaction (OER), oxygen reduction reaction (ORR), CO_2_ hydrogenation reaction, energy storage and toluene oxidation [11,12,13,14,15,16,17,18,19,20]. For a synthetic strategy of Co_3_O_4_@TiO_2_ composite, thermal decomposition of ZIF-67 followed by the liquid-phase deposition method was introduced [11]. The catalyst showed an overpotential of 269 mV at 10 mA cm^−2^ and a Tafel slope of 106 mV/dec for OER test in 2 M KOH electrolyte, and an overpotential of 153 mV and a Tafel slope of 81 mV/dec in HER test, respectively. Salahuddin et al. prepared nano-porous carbon by thermal treatment of ZIF-67, hybridized with MnO_2_, and demonstrated ORR experiments for fuel cell applications [12]. Amorphous intermediate and crystalline Co_3_O_4_ was synthesized by thermal treatment of ZIF-67 [13], and the amorphous sample showed an OER onset potential of 1.4 V (vs. reference hydrogen electrode, RHE), lower than that (1.52 V) of a crystalline sample. For MXene-supported CoNi-ZIF-67 catalysts by the coprecipitation method, the OER test showed an onset overpotential of 275 mV (vs. RHE) and a Tafel slope of 65.1 mV/dec [15]. B-Co_3_O_4_@ZIF-67 nanocages were synthesized by the hydrothermal method using a sacrificial template, and showed an overpotential of 334 mV at a current density of 10 mA cm^−2^ and a Tafel slope of 73.88 mV for OER [18]. Another work introduced bimetallic ZIF-8 and ZIF-67 to synthesize Ru-decorated Co/N-codoped porous carbon catalysts, and the HER performance showed an overpotential of 30 mV at 10 mA cm^−2^ and a Tafel slope of 32.1 mV/dec. The stability was claimed to be superior than a commercial 20 wt% Pt/C [20]. A MOF-derivatized Z-scheme catalyst of Fe_2_O_3_@Ag–ZnO@C was also introduced to photodegrade tetracyclin and methylene blue in wastewater [21]. The developed catalyst showed high light absorption and charge transfer efficiency for higher photocatalytic performance. For an application area of CO_2_ reduction [25,26,27], CdS/ZIF-67 nanocomposites were synthesized by a simple wet reaction method and tested for photocatalytic CO_2_ reduction reaction under a 300 W Xe lamp (with 420 nm cut-off filter) [23]. The catalysts showed CO and H_2_ as the main products, with 184 μmol g^−1^·h^−1^ and 1098 μmol g^−1^ h^−1^, respectively, and a CO selectivity of 5–45%.

As discussed in the literature, many strategies have been employed and the consequent materials have been tested in various application areas [1,2,3,4,5,6,7,8,9,10,11,12,13,14,15,16,17,18,19,20,21,22,23,24,25,26,27]. Motivated by this, we seek to further widen the synthesis of ZIP-derivatized catalyst materials and examine new physicochemical properties of a newly synthesized material. Thus, we prepared bean sprout-like Co-TiO_2_/Ti nanostructures by the thermal treatment of ZIF-67/Ti hybrid structure and demonstrated diverse catalytic applications of photocatalytic CO_2_ reduction, electrochemical CO_2_ reduction and electrochemical oxygen evolution reaction. Although ZIF-derivatized catalyst have been extensively studied for water splitting, few studies have been reported related to CO_2_ reduction [25,26,27]. Therefore, the present study shows some data sets for catalytic performances on CO_2_ reduction. ZIP-67 was chosen because Co is in the structure, and consequent Co oxide-based catalysts have potentially high applicability to water splitting reactions [28]. Cu and Ti were chosen because they have been extensively used as substituent materials and catalyst supports in photocatalysis and electrocatalysis [28,29,30,31,32]. Therefore, the present results provide a useful strategy for the design of new catalyst materials with high efficiency for energy and environment fields via CO_2_ reduction and water splitting.

## 2. Materials and Methods

### 2.1. Preparation of ZIF-67/Ti and Co-TiO_2_/Ti Catalysts

Chemicals were Co(NO_3_)_2_∙6H_2_O (97.0%, Samchun Pure Chem., Kyoungki, Korea), 2-methylimidazole (99%, Sigma-Aldrich, Saint Louis, MO, USA) and methanol (MeOH, 99.9%, Samchun Pure Chem., Kyoungki, Korea). For the preparation of ZIF-67/Ti, a Ti sheet was roughened by sandpaper followed by sonication in deionized water for 30 min, and then dipped in 1% HNO_3_ solution for 1 min. After that, the sheet was finally washed with deionized water and dried under an infrared (IR) lamp. For the preparation of ZIF-67/Ti, a solution of Co(NO_3_)_2_∙6H_2_O (2mmol) in 40 mL MeOH and a solution of 2-methylimidazole (20 mmol) in 40 mL MeOH were fully mixed, and then the cleaned Ti sheet was dipped in the mixed solution for 16 h to load ZIF-67. The consequent ZIF-67/Ti was then gently washed with MeOH and dried under an IR lamp. After that, the as-prepared ZIF-67/Ti sheet was placed in a tube furnace with Ar gas (99.999%) flow (80 mL/min). The furnace temperature was then increased to 800 °C with a ramp rate of 23 °C/min, and once the temperature reached 800 °C, the power was turned off, and we let the furnace naturally cool to room temperature. Finally, we obtained Co-TiO_2_/Ti nanostructures. Cu deposition on the nanostructures was performed using a SPT-20 ion sputter coater (COXEM Co., Daejeon, Korea) at an ionization current of 3 mA for 10 s, 30 s, 60 s, 120 s, 240 s, 480 s and 960 s, respectively.

### 2.2. Characterisation of the Catalyst Samples

The crystal phases of the materials formed on Ti sheets were examined using a MiniFlex II X-ray diffractometer (Rigaku Corp., Tokyo, Japan) with a Cu K_α_ radiation source (CNU Chemistry Core Facility). A VESTA software (ver. 3.5.7, free downloaded at https://jp-minerals.org/vesta/en/download.html, accessed on 30 April 2021) was employed for crystal structure building and facet visualization [33]. A FEI Tecnai G2 F30 S-TWIN TEM (FEI-Philips, Hillsboro, OR, USA) at 300 kV was used to obtain high resolution transmission electron microscope (HR-TEM) images. The morphologies of the samples were all examined using a S-4800 (Hitachi Ltd., Tokyo, Japan) scanning electron microscope (SEM) at an acceleration voltage of 10.0 keV. Energy-dispersive X-ray spectroscopy (EDXS) was employed to examine elemental compositions and mapping images using a SEM (Merlin Compact, Carl Zeiss, Germany), coupled with an AZtec Energy X-MaxN EDXS (OXFORD, Oxford, UK). A LabRAM HR-800 UV-Visible-NIR Raman spectrometer (Horiba Jobin Yvon Kyoto, Japan) was used to take Raman spectra with a laser wavelength of 514 nm and a 100× objective, and the monochromator grating of 1800. X-ray photoelectron spectra (XPS) were obtained using a K-alpha^+^ XPS spectrometer (Thermo-VG Scientific, Waltham, MA, USA) with a hemispherical energy analyzer and a monochromated Al *K*a X-ray (E = 1486.6 eV) source. Temperature-programmed reaction spectrometry was performed using a QGA quadrupole mass gas analyzer (Hiden Analytical Inc., Warrington, United Kingdom). A ZIF-67/Ti (5 mm × 30 mm) sample was loaded in a U-tube quartz reactor and heated at a temperature heating rate of 20 °C/min under N_2_ gas (99.999%) flow (80 mL/min). The gas products with temperature were real-time monitored using the QGA analyzer.

### 2.3. Photocatalytic and Electrocatalytic CO_2_ Reduction Experiments

Photocatalytic CO_2_ reduction tests were performed in a closed stainless-steel chamber (volume ~40 mL) with a quartz window. A bare (or Cu-deposited) Co-TiO_2_/Ti disc (a diameter of 47 mm) was placed inside the chamber. Before being closed, the chamber was flushed and filled with pure CO_2_ gas (99.999%) with 60 μL deionized water inside the chamber. For CO_2_ reduction test, the sample was placed under UVC light (200–280 nm, a power density of 5.94 mW/cm^2^) through the window for 6 h.

For electrochemical CO_2_ reduction experiment, a three-electrode connection system was used with a Pt counter electrode, a Ag/AgCl (3.0 M KCl) reference electrode and a Co-TiO_2_/Ti electrode (5 mm × 30 mm) working electrode. The electrochemical workstation was a WPG100 Potentiostat/Galvanostat (WonATech Co., Ltd., Seoul, Korea). We used an air-tight closed glass cell (100 mL) with 50 mL 0.1 M NaHCO_3_ electrolyte. Amperometry was performed at a potential of −1.8 V (vs. Ag/AgCl) for 3 h.

After the amperometry (or the photocatalytic reaction under UVC condition for 6 h), 0.5 mL volume of gas from the air-tight closed electrochemical cell (or the closed stainless-steel chamber) was taken and injected into a YL 6500 gas chromatography (GC) system (Young In Chromass Co., Ltd., Seoul, Korea) for the analysis of CO_2_ reduction gas products. The GC system was equipped with a Ni catalyst methanizer assembly, a thermal conductivity detector (TCD), a flame ionization detector (FID), two different columns of 40/60 Carboxen-1000 (Sigma-Aldrich, St. Louis, MO, USA) and HP-PlotQ-PT (Agilent Technologies, Inc., Santa Clara, CA, USA).

Electrochemical oxygen evolution reaction (OER) tests were also performed using a three-electrode system (a Pt counter electrode, a Hg/HgO reference electrode and a Co-TiO_2_/Ti electrode working electrode). The electrochemical workstation was a WizECM-1200 Premium potentiostat/galvanostat (WizMAC, Daejeon, Korea). Linear sweep voltammetry (LSV) was carried out at a scan rate of 20 mV/s in 0.1 M KOH electrolyte (a volume of 50 mL), with a potential range from −0.05 V to +1.5 V.

## 3. Results and Discussion

**Figure 1:**
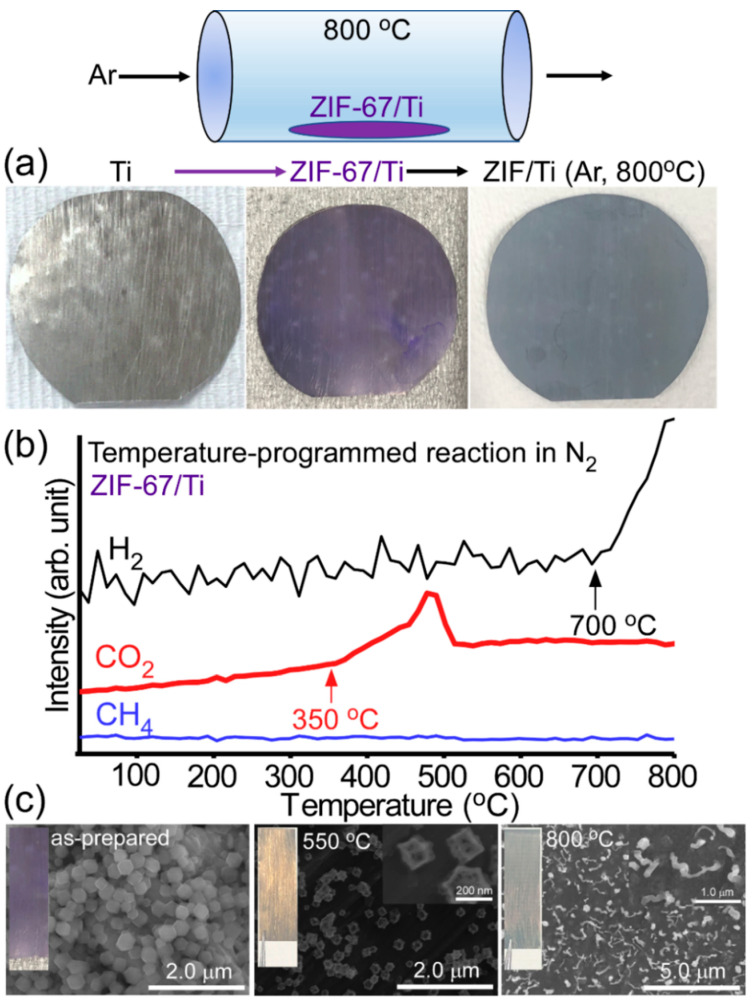
Schematic of a reaction setup and photos of the samples at each stage (**a**); temperature-programmed reaction profiles (**b**) of H_2_, CO_2_ and CH_4_ for ZIF-67/Ti sheet; and SEM images (**c**) of as-prepared ZIF-67/Ti and after thermal treatments of 550 °C and 800 °C, respectively.

Figure 1a shows the sample preparation method and photos of the samples at each stage. The polished Ti sheet appeared bright gray and became violet upon loading ZiF-67. Upon thermal treatment at 800 °C, the color finally became uniform blue-gray. This indicates that surface morphology and crystal phase became changed after the thermal treatment. Figure 1b displays temperature-programmed reaction (TPR) profiles for the as-prepared ZIF-67 on Ti sheet to examine thermal decomposition of the ZIF-67 on the Ti surface. It was observed that TPR signal of CO_2_ (mass = 44 amu) started to increase at around 350 °C, maximize at 490 °C and return to the background signal above 510 °C. This indicates that ZIF-67 decomposed at around 490 °C. This result is consistent with the literature [34]. 

The TPR signal (Figure 1b) of H_2_ (mass = 2 amu) started to increase at 700 °C, which was attributed to the association of adsorbed H, followed by H_2_ release. The CH_4_ signal showed no critical change. The corresponding SEM images (Figure 1c) at each stage (as-prepared, 550 °C and 800 °C conditions) clearly showed different morphologies. For the SEM image of the as-prepared ZIF-67/Ti, the morphology appeared to be polyhedron shape with sizes of 30–40 nm. This is consistent with the literature regarding ZIF-67 [15]. After thermal treatment up to 550 °C, ZIF-67 became shrunken and hollow, which was attributed to the thermal decomposition at 490 °C, as discussed above. Interestingly, the morphology drastically changed after thermal treatment up to 800 °C. The corresponding SEM image appeared as bean sprouts with head and stem. The head appeared to be grown from the surface of Ti surface. This is further discussed below.

**Figure 2:**
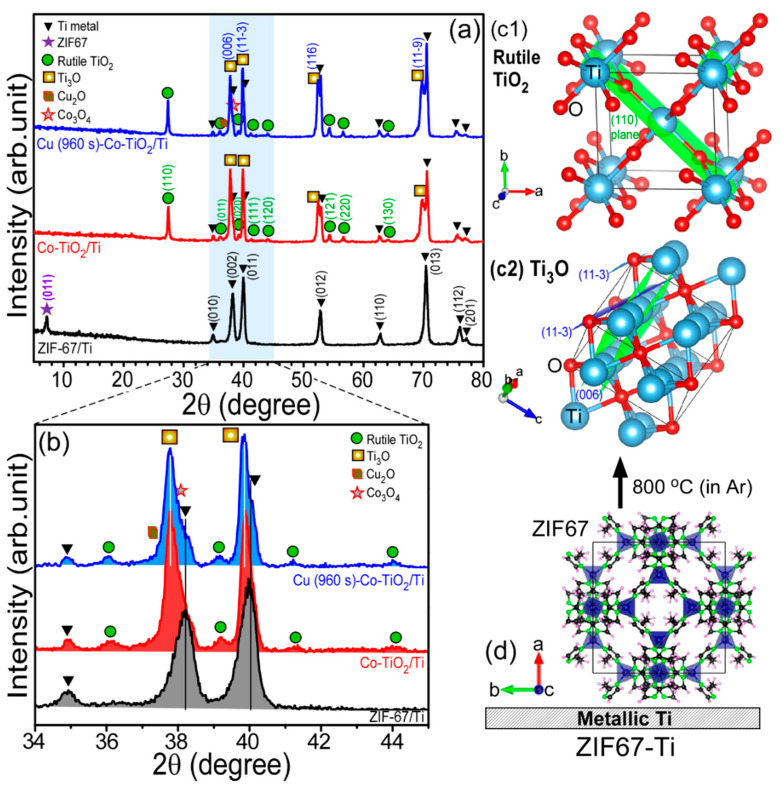
XRD diffraction patterns (**a**) of as-prepared ZIF-67/Ti, Co-TiO_2_/Ti, Cu (960 s)-Co-TiO_2_/Ti samples; expanded XRD regions (34–45°) (**b**); crystal structure projections of rutile TiO_2_ (**c1**); and Ti_3_O (**c2**); and a cartoon of ZIF67 loaded on Ti support (**d**).

Three samples of ZIF-67/Ti, Co-TiO_2_/Ti, Cu(960 s)-Co-TiO_2_/Ti were selected, and their XRD profiles were obtained and displayed in Figure 2. For the XRD patterns of a ZiF-67 loaded Ti sheet, several major peaks were observed at 2 θ = 35.2°, 38.5°, 40.2°, 53.1°, 63.1° and 70.8°, assigned to the (010), (002), (011), (012), (110) and (013) planes of hexagonal metallic Ti (ICSD ref. # 98-004-3614), respectively. These speaks were commonly observed for all the samples attributed to metallic Ti support. A sharp peak around 2 θ = 8.5° was attributed to the crystal phase of ZIF-67 [13,14,15]. For the XRD patterns of Co-TiO_2_/Ti sample, several peaks newly appeared at 2 θ = 27.4°, 36.0°, 39.1°, 41.2°, 44.0°, 54.2°, 56.5° and 63.9°, and were assigned to the (110), (011), (020), (111), (120), (121), (220) and (130) crystal planes of tetragonal rutile TiO_2_ (ICSD ref. # 98-003-3838), respectively. Other strong peaks were observed at 2 θ = 29.1°, 39.8°, 52.3° and 69.5°, and matched the crystal plans of (006), (11-3), (116) and (11-9) for hexagonal Ti_3_O (ICSD ref. # 98-003-6055), respectively. The expanded XRD profiles between 2 θ = 34° and 45° clearly show that metallic Ti and hexagonal Ti_3_O coexist in the thermal (800 °C)-treated samples [35]. The XRD patterns of Co and Cu oxides were hardly seen in the XRD profiles. This is further discussed below.

**Figure 3:**
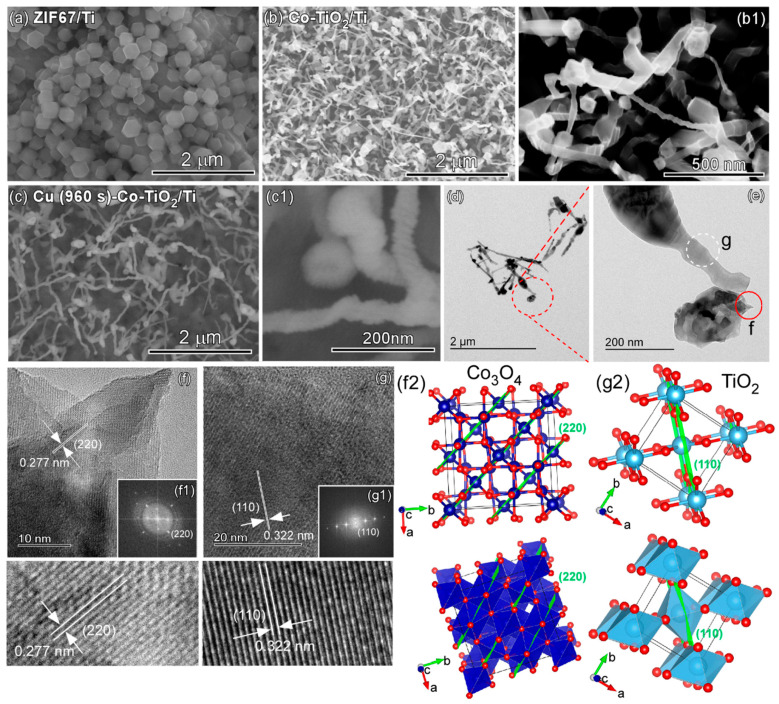
SEM images of ZIF67-Ti (**a**), Co-TiO_2_/Ti (**b**,**b1**), Cu (960 s)-Co-TiO_2_/Ti (**c**,**c1**). TEM (**d**,**e**) and HRTEM (**f**,**g**) images of Co-TiO_2_/Ti. Insets show the corresponding FFT patterns (**f1**,**g1**) and the structure projection (**f2**,**g2**) of the (220) and (110) planes for Co_3_O_4_ (**f2**) and rutile TiO_2_ (**g2**), respectively.

Figure 3 shows the SEM images of the as-prepared ZIF-67/Ti (Figure 3a) and Co-TiO_2_/Ti (Figure 3(b,b1)) and Cu (960 s)-Co-TiO_2_/Ti (Figure 3(c,c1)) samples. As discussed above, the polyhedron shape in Figure 3a was due to ZIF-67, and was commonly reported in the literature [15]. For the SEM image of Co-TiO_2_/Ti, the morphology appeared to be bean sprouts with head and stem. For Cu (960 s)-Co-TiO_2_/Ti (Figure 3(c,c1)) samples, the head and the stem appeared to be fully covered by Cu. For the TEM images of the bean sprout nanostructures in Figure 3d,e, two regions (f = head and g = stem) were selected to obtain HRTEM images. For the HRTEM image of the head (Figure 3f), clear lattice firings were seen with a distance of 0.277 nm. This is in good agreement with the lattice spacing of the (220) plane of cubic phase Co_3_O_4_ [36]. The corresponding fast-Fourier-transform (FFT) pattern (Figure 3(f1)) shows high crystallinity of the head. For the HRTEM image of the stem (Figure 3g), a clear lattice spacing of 0.322 nm was observed, and the corresponding FFT pattern (Figure 3(g1)) showed high single crystalline nature of the stem. The lattice spacing was consistent with the (110) plane of tetragonal rutile TiO_2_ [37], which was observed by the XRD, discussed above. For visual understating of the crystal phase and the facet, the crystal structure projections with the crystal planes are displayed in Figure 3(f2,g2) for Co_3_O_4_ and TiO_2_, respectively. 

**Figure 4:**
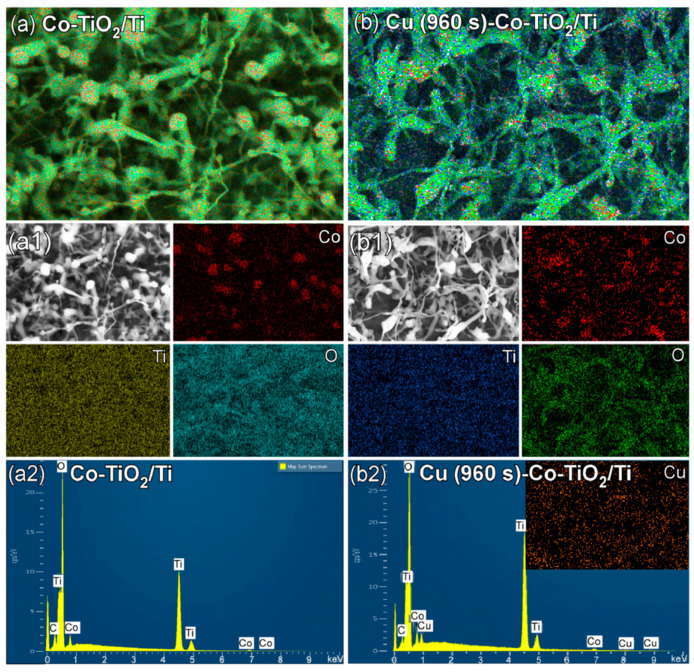
Layered (**a**,**b**) and elemental (Co, Ti, O and Cu (**a1**,**b1**)) mapping images and EDXS profiles (**a2**,**b2**) of Co-TiO_2_/Ti (**a**,**a1**,**a2**) and Cu (960 s)-Co-TiO_2_/Ti (**b**,**b1**,**b2**).

Figure 4 shows layered and single elemental (Co, Ti, O, and Cu) EDXS mapping images and EDXS profiles of Co-TiO_2_/Ti and Cu (960 s)-Co-TiO_2_/Ti samples. As discussed above in the above SEM images (Figure 3(b,b1,c,c1)) and the SEM images in Figure 4(a1,b1), Co-TiO_2_/Ti nanostructures appeared to be bean sprouts with head and stem. To confirm the chemical elements of the head and stem, elemental EDXS mapping images were obtained. On the basis of the images, Co EDXS signal was mainly localized on the head, indicating that the head was mainly due to Co elements while others were mainly due to Ti oxides. This is in good agreement with the TEM/HRTEM results discussed above. The Co and Ti oxide were originated from the ZIP-67 and Ti support, respectively. Upon Cu-deposition, Cu EDXS signal was newly detected. The EDXS profiles (Figure 4(a2,b2)) clearly confirmed the elements of Ti, Co, surface C and Cu (after Cu-deposition), Ti (0.395, 0.452, 4.511, and 4.932 keV), O (0.525 keV), C (0.277 keV), Co (0.678, 0.694, 0.776, 6.93 and 7.65 keV) and Cu (0.811, 0.832, 0.93, 8.048 and 8.905 keV) [38]. For the Co-TiO_2_/Ti sample, the atomic % ratios of C, Ti O, and Co were estimated to be 3.7%, 28.9%, 64,8% and 2.6%, respectively. The Co/Ti ratio (%) was estimated to be 2.6%/28.9% = 9.0/100. For the Cu (960 s)-Co-TiO_2_/Ti sample, the atomic % ratios of C, Ti, O, Co and Cu were estimated to be 2.6%, 35.2%, 58.6%, 2.5% and 1.0%, respectively. The Co/Ti ratio (%) was estimated to be 2.5%/35.2% = 7.1/100.

**Figure 5:**
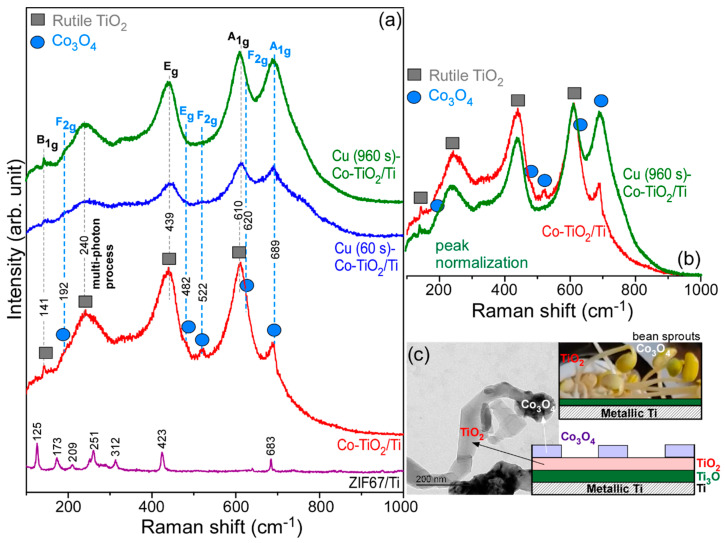
Raman spectra (**a**) of ZIF-67/Ti, Co-TiO_2_/Ti, Cu (60 s)-Co-TiO_2_/Ti and Cu (960 s)-Co-TiO_2_/Ti corresponding to normalized Raman spectra (**b**) before and after Cu deposition, and a schematic (**c**) of the material growth mode on metallic Ti surface upon thermal treatment.

Figure 5 displays the Raman spectra for ZIF-67/Ti, Co-TiO_2_/Ti, Cu (60 s)-Co-TiO_2_/Ti and Cu (960 s)-Co-TiO_2_/Ti samples. For the spectrum of ZIF-67/Ti, several sharp peaks were observed at 125 cm^−1^, 173 cm^−1^, 209 cm^−1^, 251 cm^−1^, 312 cm^−1^, 423 cm^−1^ and 683 cm^−1^. These peaks are attributed to ZIF-67 commonly reported in the literature [39]. For the Raman spectrum of a Co-TiO_2_/Ti sample, very broad and strong Raman peaks were observed at 270 cm^−1^, 439 cm^−1^ and 610 cm^−1^, in addition to weaker and sharper Raman peaks at 141 cm^−1^, 192 cm^−1^, 482 cm^−1^, 522 cm^−1^, 620 cm^−1^ and 689 cm^−1^. Three characteristic Raman active peaks at 141 cm^−1^, 439 cm^−1^ and 610 cm^−1^ could be assigned to the B_1g_, E_g_ and A_1g_ modes of rutile TiO_2_, respectively [40,41]. The corresponding peak at 240 cm^−1^ is due to the multi-phonon scattering process [41]. In addition to the Raman peaks of TiO_2_, five other Raman active peaks were observed at 192 cm^−1^, 482 cm^−1^, 522 cm^−1^, 620 cm^−1^ and 689 cm^−1^, assigned to F_2g_, E_g_, F_2g_, F_2g_ and A_1g_ modes of Co_3_O_4_, respectively [42,43]. On the basis of the results, it was concluded that the major was TiO_2_ (the stem) and minor was Co_3_O_4_ (the head) in the bean sprout nanostructures. 

Upon Cu deposition for 60 s, no characteristic of Cu species was observed. However, interestingly, the A_1g_ mode at 689 cm^−1^ was substantially increased relative to the other Raman peaks. Furthermore, in the normalized Raman peaks of ZIF-67/Ti (red line) and Cu (960 s)-Co-TiO_2_/Ti (green line) samples (Figure 5b), the A_1g_ mode became substantially enhanced after Cu deposition for 960 s. The Raman peaks for the Cu species were not clearly detected [44,45]. As depicted in Figure 5c, it may appear that Ti_3_O was formed at the interface of Ti and TiO_2_, and Co_3_O_4_ was grown as a head during thermal deposition of ZIF-67, as discussed in Figure 1b above.

**Figure 6:**
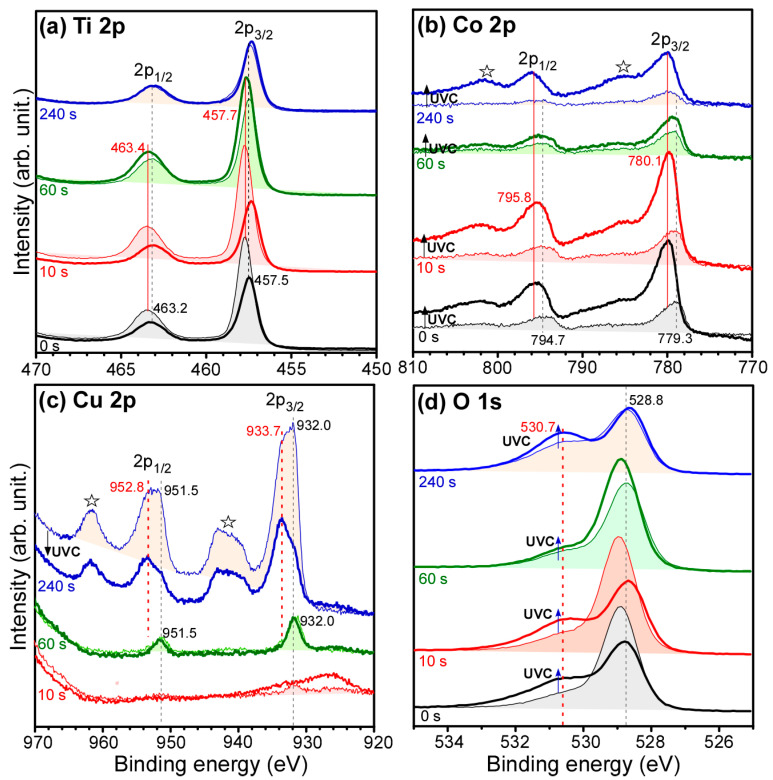
Ti 2p (**a**), Co 2p (**b**), Cu 2p (**c**) and O 1s (**d**) XPS profiles for bare Co-TiO_2_/Ti and 10s, 60 s and 240 s-Cu-deposited Co-TiO_2_/Ti samples before (thin lines) and after (thick lines) UVC photocatalytic reaction. ☆ I ndicates shake-up structures.

Figure 6 displays Ti 2p (Figure 6a), Co 2p (Figure 6b), Cu 2p (Figure 6c) and O 1s (Figure 6d) XPS profiles for bare Co-TiO_2_/Ti and Cu deposited (10 s, 60 s and 240 s) Co-TiO_2_/Ti samples before and after UVC photocatalytic CO_2_ reduction tests. The elements of Ti, Co, C and O were commonly observed as expected, and the Cu element was additionally observed after Cu deposition. On the basis of the Ti 2p and Co 2p XPS intensities and sensitivity factors, the Co/Ti XPS ratio was estimated to be 5.3/100 for bare Co-TiO_2_/Ti. The Co/Ti XPS ratio became 3.7/100 after Cu deposition for 240 s. As discussed above, the decrease in Co/Ti was also observed in the EDXS data, which is consistent with the XPS data. This indicates that the head of Co is covered more by Cu compared to the stem of Ti.

For the Ti 2p XPS profiles of bare Co-TiO_2_/Ti (Figure 6a), the Ti 2p_3/2_ and Ti 2p_1/2_ peaks were observed at binding energies (BEs) of 457.7 eV and 463.4 eV, respectively, with a spin-orbit (S-O) splitting energy of 5.7 eV. These peaks were attributed to Ti(IV) state of TiO_2_ [30]. The Ti 2p BE peaks showed no critical change with an increasing Cu deposition time, but the intensity was somewhat decreased due to overlayer Ti. Furthermore, the asymmetry of the Ti 2p peak was increased with an increasing Cu deposition (Supplemental Information, Figure S1a). The Ti 2p XPS signal appeared to increase around 459 eV and 465 eV. The asymmetry of Ti 2p XPS peak could be a change in either chemical state or physical nature [46].

In Figure 6b, the corresponding Co 2p_3/2_ and Co 2p_1/2_ peaks were observed at 779.3 eV and 794.7 eV, respectively, with an S-O splitting energy of 15.4 eV. These peaks could be assigned to Co_3_O_4_, as expected from the HRTEM and Raman spectra. The Co 2p peak showed no substantial change with increasing Cu deposition time based on the normalized Co 2p XPS spectra with Cu deposition time (Supplemental Information, Figure S1b), but the intensity decreased because of the overlayer Cu.

The corresponding O 1s XPS peaks (Figure 6d) showed two broad peaks 528.8 eV and 530.7 eV, attributed to lattice oxygen (O_lattice_) of metal oxide and surface oxygen species (e.g., O_ad_: OH/H_2_O and defects), respectively [30]. With increasing Cu deposition time, the higher BE peak at 530.7 eV was observed to be enhanced (Supplemental Information, Figure S1c), which was attributed to increase in surface oxygen species. For the bare Co-TiO_2_/Ti sample, the XPS O_ad_/O_lattice_ ratio was measured to be 17.4%/82.6% and the ratios became 16.0%/84.0%, 24.7%/75.2% and 50.5%/49.5% after Cu deposition for 10 s, 60 s and 240 s, respectively. 

For the Cu 2p XPS profile of Cu-deposited (10 s and 60 s) Co-TiO_2_/Ti samples (Figure 6c), the Cu 2p_3/2_ and Cu 2p_1/2_ peaks were commonly observed at 932.0 eV and 951.5 eV, respectively, with an S-O splitting energy of 19.5 eV. These peaks could be assigned to Cu_2_O and/or metallic Cu [46,47,48]. For the bare Co-TiO_2_/Ti sample, no Cu XPS signal was observed, as expected. For the Cu 2p XPS profile of Co-TiO_2_/Ti sample after Cu-deposition for 240 s, additional Cu 2p_3/2_ and Cu 2p_1/2_ peaks were observed at 933.7 eV and 952.8 eV, respectively, with an S-O splitting energy of 19.1 eV. Moreover, broad shake-up structures (☆) were newly observed around 942 eV and 962 eV. These new Cu 2p peaks and the characteristic shake-up features have commonly been attributed to CuO (cupric oxide) [46,47,48].

For the XPS profiles after photocatalytic CO_2_ reduction tests, Ti 2p peaks of bare and Cu (10 s)-Co-TiO_2_/Ti samples were decreased, while the Co 2p signal was relatively increased. At higher Cu coverages of 60 s and 240 s, Ti 2p signal was enhanced, as was the Co 2p signal. The normalized Ti 2p peaks showed no critical difference with Cu thickness after CO_2_ reduction (Supplemental Information, Figure S1(a1–a3). However, for the Co 2p XPS of Cu (240 s)-Co-TiO_2_/Ti sample (Figure 6b), satellite peaks at 786 eV and 802 eV were enhanced after the photocatalytic CO_2_ reduction, which was attributed to an increase in CoO state (Supplemental Information, Figure S1(b1–b3) [15,27,43,48]. For the O 1s XPS profiles, the XPS O_ad_/O_lattice_ ratios were substantially changed after the photocatalytic CO_2_ reduction. The O_ad_/O_lattice_ ratios were 51.9%/48.1%, 49.2%/50.8%, 19.6%/80.4% and 53.9%/46.1% for bare, Cu(10 s)-, Cu (60 s)- and Cu (240 s)-Co-TiO_2_/Ti samples, respectively. Except for the Cu (60 s)-Co-TiO_2_/Ti sample, the O_ad_ XPS peak was significantly enhanced after the photocatalytic CO_2_ reduction (Supplemental Information, Figure S1(c1)). For the Cu 2p XPS profile of the Cu (240 s)-Co-TiO_2_/Ti sample after the reduction test, the intensity was somewhat decreased (Supplemental Information, Figure S1d), which was possibly due to agglomeration of smaller Cu particles forming larger Cu particles. In addition, the Cu 2p_3/2_ (Cu 2p_1/2_) peak at 933.7 eV (952.8 eV) became stronger than the peaks at 932.0 eV (951.5 eV). This is mainly due to CuO species being more present than Cu_2_O species. Carbon signals were somewhat increased both at 284 eV and 288 eV after photocatalytic CO_2_ reduction (Supplemental Information, Figure S1e), which was attributed to an increase in C–C and C=O species.

**Figure 7:**
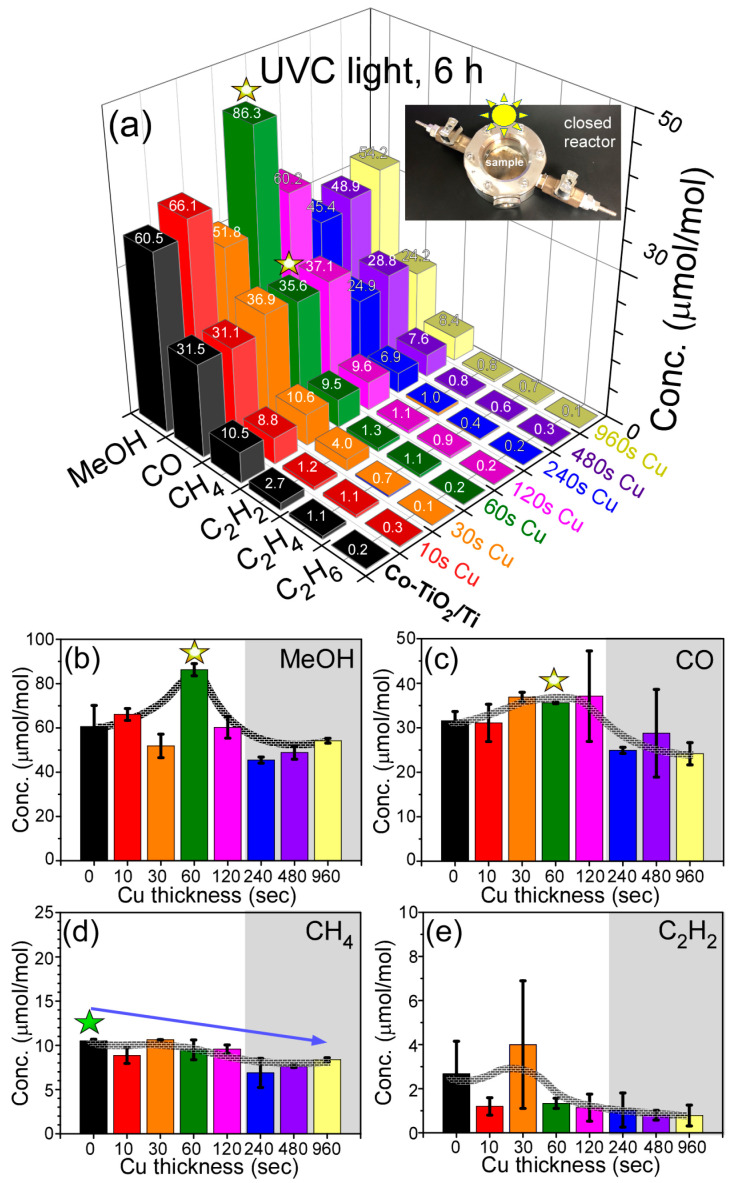
Photocatalytic CO_2_ reduction yields: (**a**) for bare Co-TiO_2_/Ti and 10s, 60 s, 120 s, 240 s, 480 s and 960 s-Cu-deposited Co-TiO_2_/Ti samples, and MeOH (**b**), CO (**c**), CH_4_ and (**d**) C_2_H_2_ (**e**) yields (μmol/mol) with error bars. Photo is a closed stainless reactor with a Co-TiO_2_/Ti disc inside. The asterisk indicates the maximum point.

Photocatalytic CO_2_ reduction experiments [30,48,49,50,51,52] were performed, and the amounts in the reduction products are displayed in Figure 7a. The products were commonly observed to be MeOH, CO, CH_4_, C_2_H_2_, C_2_H_4_ and C_2_H_6_. The yields (μmol/mol = ppm) of the three major products commonly showed the order of CH_4_ < CO < MeOH. For the minor products, C_2_H_2_ was also detected in the range of 1.0–4.0 ppm. Although the production amounts were below 1 ppm, C_2_H_4_ and C_2_H_6_ were meaningfully detected in the gas chromatography (GC) profiles. No H_2_ was detected in the GC profile. Choi et al. performed a photocatalytic CO_2_ reduction test for a bare Co_3_O_4_ powder sample under the same experimental conditions as in the present study, except for a reaction time of 13 h vs. 6 h [52]. They reported that three major products showed the order of CH_4_ (17.1 ppm) < MeOH (19.1 ppm) < CO (83.2 ppm) for 13 h. Lin et al. reported CO as a major photocatalytic CO_2_ reduction product for [Co(bipy)_3_]^2+^-embedded TiO_2_ hollow spheres [53]. On the basis of the literature and the present study, MeOH production was highly enhanced in the developed Co-TiO_2_/Ti nanostructure.

For the bare Co-TiO_2_/Ti sample, MeOH, CO, CH_4_, C_2_H_2_, C_2_H_4_ and C_2_H_6_ were observed to be 60.5 ppm, 31.5 ppm, 10.5 ppm, 2.7 ppm, 1.1 ppm and 0.2 ppm. MeOH production (Figure 7b) showed the highest production of 86.3 ppm after Cu deposition for 60 s. Above Cu deposition of 240 s, MeOH production was observed to be lower than that in the bare Co-TiO_2_/Ti sample. CO production (Figure 7c) was somewhat increased upon Cu deposition for 30 s–120 s. Upon Cu deposition above 240 s, CO production was also observed to be lower than that in the bare Co-TiO_2_/Ti sample. For CH_4_ production (Figure 7d), the bare Co-TiO_2_/Ti sample showed the highest amount of 10.5 ppm, and the yield was gradually decreased with an increasing Cu deposition amount. For C_2_H_2_ production (Figure 7e), the amount was higher when Cu amount was below 30 s, but the amount dropped below 1 ppm when Cu deposition was above 60 s. CO_2_ reduction selectivities were estimated to be 50–64%, 27–35% and 7–10% for MeOH, CO and CH_4_, respectively. Details are shown in the Supplemental Information, Figure S2. The 60 s-Cu-deposited Co-TiO_2_/Ti sample showed the highest selectivity of 64% for photocatalytic MeOH production.

Conclusively, photocatalytic CO_2_ reduction activity became evidently poorer upon Cu deposition above 240 s, which was attributed to less light absorption by thicker Cu film. However, Cu deposition between 30 s and 120 s showed meaningful enhancement in CO_2_ reduction activity. 

**Figure 8:**
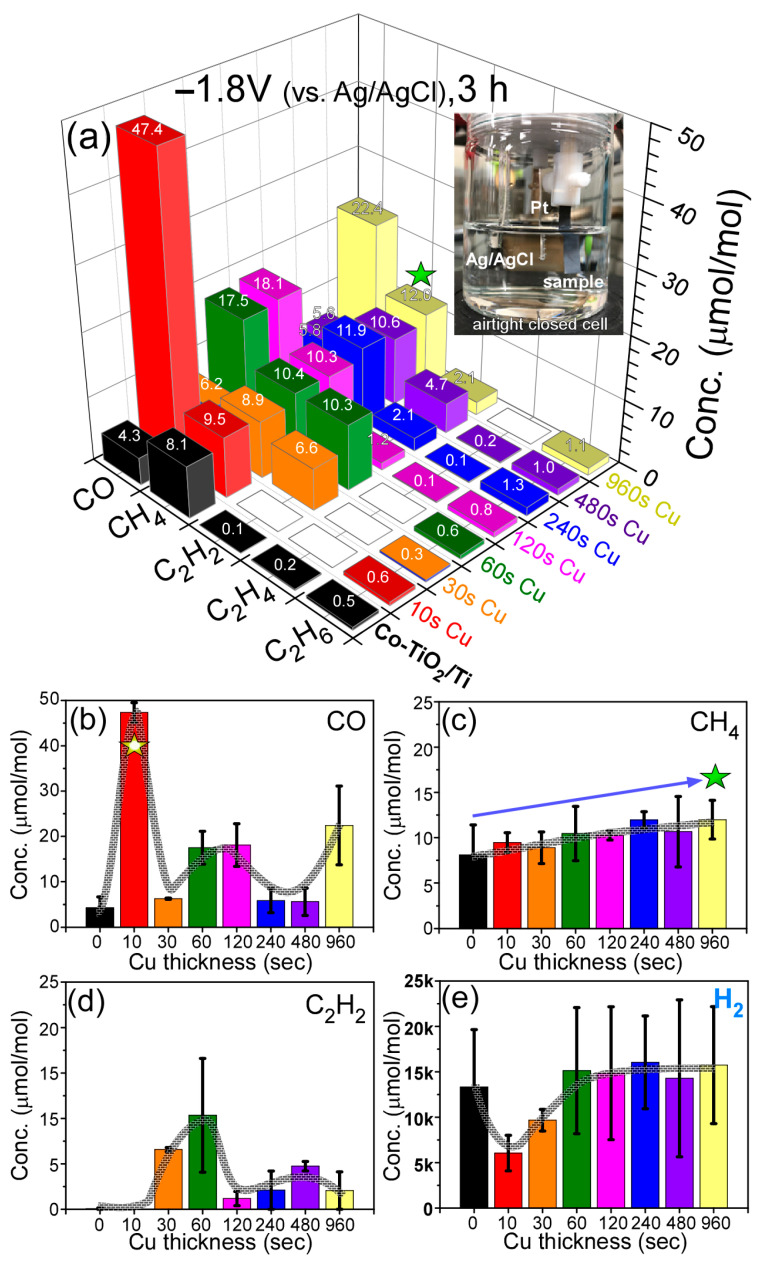
Electrocatalytic CO_2_ reduction product yields (**a**) for bare Co-TiO_2_/Ti and 10s, 30 s, 60 s, 120 s, 240 s, 480 s and 960 s-Cu-deposited Co-TiO_2_/Ti samples, and CO (**b**), CH_4_ (**c**), C_2_H_2_ (**d**) and H_2_ (**e**) yields (μmol/mol) with error bars. Photo is the electrochemical 3-electrode air-tight closed cell. The asterisk indicates the maximum point for CH_4_.

Electrochemical CO_2_ reduction experiments [30,49,54,55] were also performed, and the amounts of reduction products are displayed in Figure 8a. In this experimental setup, gaseous products were only measured by GC and no MeOH was detected in the gas phase. The reproducibly detected gaseous products include CO, CH_4_, C_2_H_2_, C_2_H_4_ and C_2_H_6_. The amounts were much less than 50 ppm. However, H_2_ was significantly detected with the amounts up to 13,325 ppm. Faradaic efficiency (FE, %) for H_2_ production was estimated to be about 6–10% in the NaHCO_3_ electrolyte condition. CO and CH_4_ productions over the bare Co-TiO_2_/Ti sample were observed to be 4.3 ppm and 8.1 ppm, and C_2_H_2_, C_2_H_4_ and C_2_H_6_ were merely detected with 0.1 ppm–0.5 ppm. 

When Cu was sputter-deposited for 10 s, CO was substantially increased to 47.4 ppm (Figure 8b); this is an 11× increase compared with that in the bare Co-TiO_2_/Ti sample. Upon Cu deposition above 30 s, the CO production was less than that for the Cu (10 s)-Co-TiO_2_/Ti sample, but higher than in the bare Co-TiO_2_/Ti sample. In Figure 8c, the CH_4_ production was gradually increased with an increasing Cu deposition, reaching 12.0 ppm upon Cu deposition for 960 s. Inversely, as discussed above (Figure 7d), CH_4_ production decreased with increasing Cu deposition in the photocatalytic CO_2_ reduction. For C_2_H_2_ production in Figure 8d, the production was ≤0.1 ppm when the Cu deposition was less than 10 s. However, the amounts were increased and detected with 6.6 ppm and 10.3 ppm upon Cu deposition for 30 s and 60s, respectively. Above 60 s and up to 960 s, the amounts were somewhat decreased and detected with 1.2 ppm–4.7 ppm. The C_2_H_4_ productions were less than 0.2 ppm for all samples. C_2_H_6_ was meaningfully detected in the range of 0.5–1.3 ppm and generally increased with increasing Cu amounts. H_2_ was more substantially produced, as mentioned above. Above 60 s and up to 960 s, H_2_ productions were detected with 14,275 ppm–15,732 ppm with FE (%) of 6.5–7.5%. H_2_ production was dominant and all the catalysts showed an H_2_ production selectivity of >99%. For the C_n_ compounds with a total of less than 1% selectivity, the relative selectivities are shown in the Supplemental Information, Figure S3.

We also tested the effect of applied potential for a selected sample of Cu (120 s)-Co-TiO_2_/Ti (Supplemental Information, Figure S4). Interestingly, H_2_ production was increased by 4.6× at −1.8 V compared with that at −1.5 V (vs. Hg/HgO). However, CO and C_2_H_2_ productions were higher at −1.5 V (vs. Hg/HgO). CO was detected with 30.4 ppm and 18.1 ppm at −1.5 V and −1.8 V, respectively. C_2_H_2_ was detected with 6.7 ppm and 1.2 ppm at −1.5 V and −1.8 V, respectively.

In the CO_2_ reduction reaction [32,49], productions of C_n_ products and H_2_ are competitively observed. When the electrode in an aqueous electrolyte, H_2_ production is expected to be significant on the electrode surface via the association (H_ad_ + H_ad_ → H_2_) of adsorbed H atoms or H_ad_ + H_3_O^+^ + e^−^ → H_2_ + H_2_O [30], as observed in the present experi byment. The H_ad_ is known to be formed via H^+^ + e^−^ → H_ad_, where H_2_O → H^+^ + OH^−^ [30]. CO_2_ reduction is a multielectron process, where electrons are either provided by photogeneration or are applied by potential electrochemistry. The generally important mechanism is demonstrated below [28,45].

Co-TiO_2_/Ti + hν → Co-TiO_2_/Ti (VB, h^+^) + Co-TiO_2_/Ti (CB, e^−^) under UV light

H_2_O + h^+^ → OH + H^+^

OH + H_2_O + 3h^+^ → O_2_ + 3H^+^

CO_2_ + 2H^+^ + 2e^−^ → CO + H_2_O

CO_2_ + 8H^+^ + 8e^−^ → CH_4_ + 2H_2_O

CO_2_ + 6H^+^ + 6e^−^ → CH_3_OH + H_2_O

2CO_2_ + 10H^+^ + 10e^−^ **→** C_2_H_2_ + 4H_2_O

The production of C_2_ compounds requires more electrons that appear to be dependent on overlayer Cu. This needs to be studied further.

**Figure 9:**
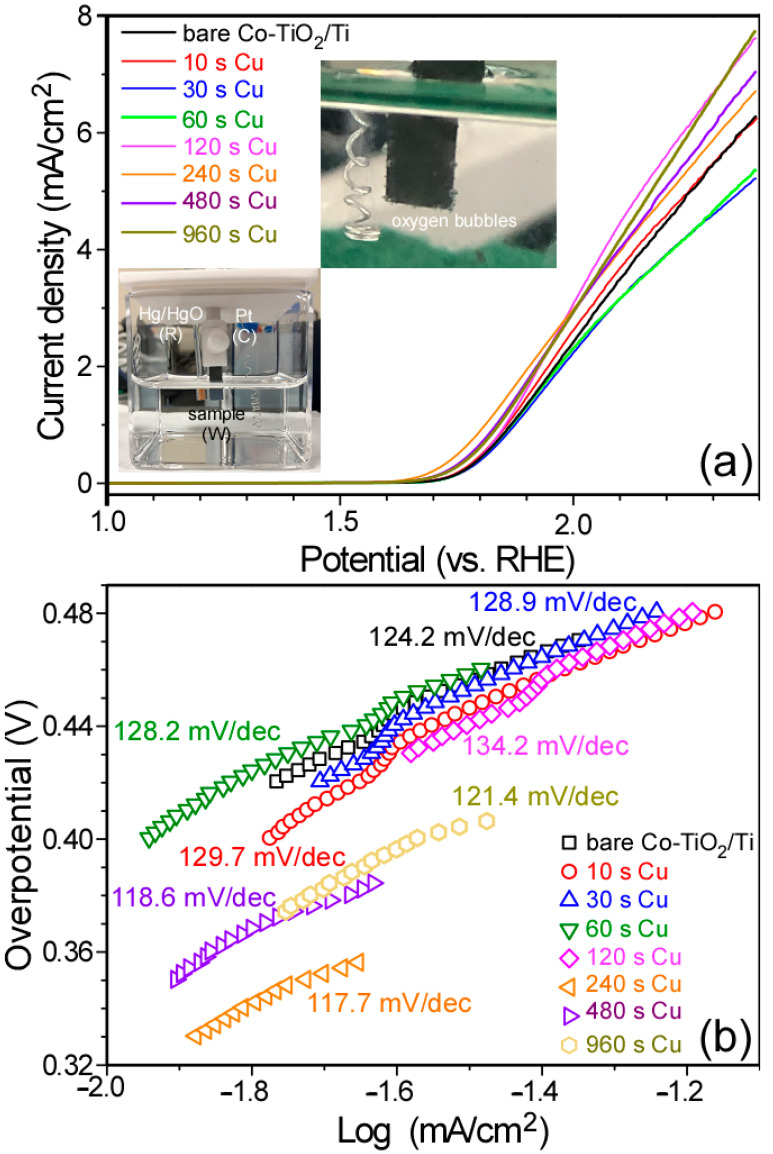
Linear sweep voltammetry profiles (**a**) for bare Co-TiO_2_/Ti and 10s, 30 s, 60 s, 120 s, 240 s, 480 s and 960 s-Cu-deposited Co-TiO_2_/Ti samples and the corresponding Tafel plots (**b**). Inset photos show the electrochemical cell with the three electrodes and oxygen bubbles on a catalyst surface.

Electrochemical oxygen evolution reaction (OER) was also performed to further extend the application area of the bean sprout-like Co-TiO_2_/Ti nanostructures. As observed above in the electrochemical CO_2_ reduction tests, H_2_ was significantly produced in the NaHCO_3_ electrolyte media, indicating that the Co-TiO_2_/Ti nanostructure had a high potential applicability to water splitting. Figure 9a displays linear sweep voltammetry (LSV) curves between 1.0 V and 2.4 V (vs. RHE) in 0.1 M KOH electrolyte at a scan rate of 20 mV/s. The X-axis was rescaled from E_Hg/HgO_ using E_RHE_ = E_Hg/HgO_ + 0.059pH + E_0_,_Hg/HgO_, where E_Hg/HgO_ is the measured potential and E_0_,_Hg/HgO_ = 0.098 V [56].

For the bare Co-TiO_2_/Ti sample, the OER onset potential was observed at around +1.65 V (vs. RHE). The onset potential showed no significant difference, with only <+0.02 V when Cu deposition was lower than 120 s. When Cu was deposited for 240 s, 480 s and 960 s, the onset potentials were observed to be +1.57 V, +1.59 V and +1.59 V (vs. RHE), respectively. These are in good agreement with the literature for ZIF-67-derivatized catalysts and Co (and/or Ni) oxide-based catalysts [13,28]. On the basis of the onset potentials (E_RHE_) from the LSV curves (Figure 9a), the OER overpotential (η) was calculated using η = E_RHE_ −1.23 V [11]. The onset overpotential was then calculated to be between 340 mV and 430 mV. The Tafel plot was obtained using the equation: η = a + b log A, where ƞ is the overpotential, a is the intercept, b is the Tafel slope and A is the current density (mA/cm^2^). As shown in Figure 9b, the Tafel slopes were estimated to be 124.2 mV/dec and 117.7 mV/dec for bare and 240 s Cu-deposited Co-TiO_2_/Ti nanostructures, respectively. When the Cu was deposited for 240 s, the OER performance was observed to be further enhanced.

**Figure 10:**
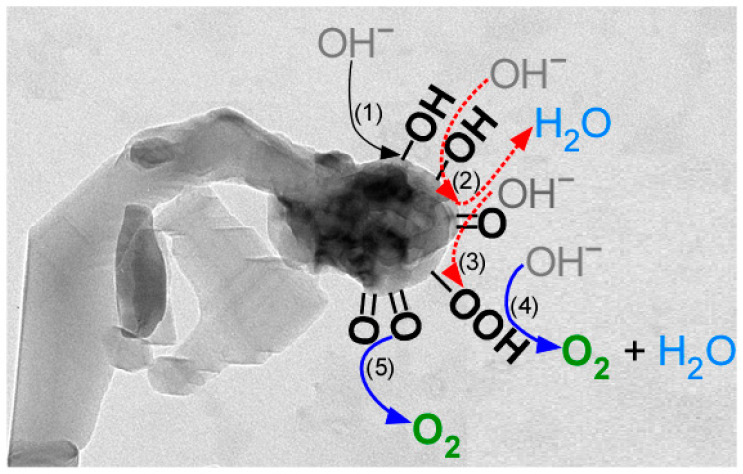
OER mechanism occurring on the Co oxide head in the bean sprout-like Co-TiO_2_/Ti nanostructures.

In the OER, the Co oxide head in the bean sprout-like structure may play a more important role in enhancing activity, as based on the literature and the findings of the present study [15,18,20,28]. The overall OER is written as M + 4OH^−^ → M + O_2_ (g) + 2H_2_O + 4e^−^ [28]. The elementary reactions are included below and the scheme is depicted in Figure 10. All these OER processes appeared to occur more efficiently on the Co oxide head. The detailed roles of the Co oxide head and TiO_2_ stem need to be investigated further.

In (1), M + OH^−^ → M – OH + e^−^

In (2), M − OH + OH^−^ → M = O + H_2_O + e^−^

In (3), M = O + OH^−^ → M − OOH + e^−^

In (4) MOOH + OH^−^ → M + H_2_O + O_2_ (↑) + e^−^

In (5), M = O + M = O → M + M + O_2_ (↑)

## 4. Conclusions

In summary, we have synthesized bean sprout-like Co-TiO_2_/Ti nanostructures using sacrificial ZIF-67 templated on Ti sheets by thermal treatment at 800 °C under Ar-flow conditions. The physicochemical properties of the newly synthesized nanostructures were examined by X-ray diffraction analysis, scanning electron microscopy, high-resolution electron microscopy, Raman spectroscopy, energy-dispersive X-ray spectroscopy and X-ray photoelectron spectroscopy. On the basis of the results, the head and the stem were observed to be Co_3_O_4_ and rutile TiO_2_, respectively. The interface of Ti support and TiO_2_ appeared to form a Ti_3_O crystal phase. Photocatalytic and electrochemical CO_2_ reduction experiments were demonstrated, as well as electrochemical OER for bare and Cu-deposited Co-TiO_2_/Ti nanostructures. The catalytic performances were observed to be dependent on the Cu deposition amount. In the photocatalytic CO_2_ reduction, GC confirmed that CH_3_OH, CO and CH_4_ were major products, with yields of 45–86 μmol/mol, 24–37 μmol/mol and 7–11 μmol/mol, respectively. Minor C_2_ compounds of C_2_H_2_, C_2_H_4_ and C_2_H_6_ were meaningfully detected under 5 μmol/mol. The highest selectivity of 64% for photocatalytic MeOH production was achieved for 60 s-Cu-deposited Co-TiO_2_/Ti sample. For electrocatalytic CO_2_ reduction, CO, CH_4_ and C_2_H_4_ were meaningfully produced under 50 μmol/mol, but H_2_ was dominantly produced with 6000 to 16,000 μmol/mol. The bean sprout-like Co-TiO_2_/Ti nanostructures were observed to show high OER performances with onset overpotentials of 330–430 mV (vs. RHE), Tafel slopes of 117–134 mV/dec and the highest performance at Cu deposition for 240 s. The present demonstration tests using ZIF-67 as a sacrificial template on metallic Ti sheet provide valuable information about the synthesis of new nanostructures, as well as potential applications regarding CO_2_ reduction and OER for energy and the environmental management.

## Data Availability

The data presented in this study are available in the article and Supplementary Material.

## Supplementary Materials

The following are available online at https://www.mdpi.com/article/10.3390/nano11081904/s1, Figure S1: XPS profiles, Figure S2: Photocatalytic CO_2_ reduction selectivities, Figure S3: Electrocatalytic CO_2_ reduction selectivities, Figure S4: Electrochemical CO_2_ reduction product yields.
