# Supplementary material for: Photocatalytic and Electrocatalytic Properties of Cu-Loaded ZIF-67-Derivatized Bean Sprout-Like Co-TiO2/Ti Nanostructures
Source: Nanomaterials (Basel). 2021 Jul 24;11(8):1904. doi: 10.3390/nano11081904 (PMC8399894; doi:10.3390/nano11081904)
Supplement: Supplementary file 1 [file nanomaterials-11-01904-s001.zip › nanomaterials-1307411-supplementary.pdf]

nanomaterials

MDPI

Supplemental Information

Photocatalytic and Electrocatalytic Properties of Cu-Loaded ZIF-67-Derivatized Bean Sprout-Like Co-TiO_2/Ti Nanostructures

Hye Ji Jang [1,2], So Jeong Park [1], Ju Hyun Yang [1,2], Sung-Min Hong [1,2], Choong Kyun Rhee [1] and Youngku Sohn [1,2,*]

[1] Department of Chemistry, Chungnam National University, Daejeon 34134, Korea; gpwldndud@naver.com (H.J.J.); jsjs5921@naver.com (S.J.P.); mil03076@naver.com (J.H.Y.); qwqe212@naver.com (S.-M.H.); ckrhee@cnu.ac.kr (C.K.R.)

[2] Department of Chemical Engineering and Applied Chemistry, Chungnam National University, Daejeon 34134, Korea

* Correspondence: youngkusohn@cnu.ac.kr; Tel.: +82-(42)-8216548

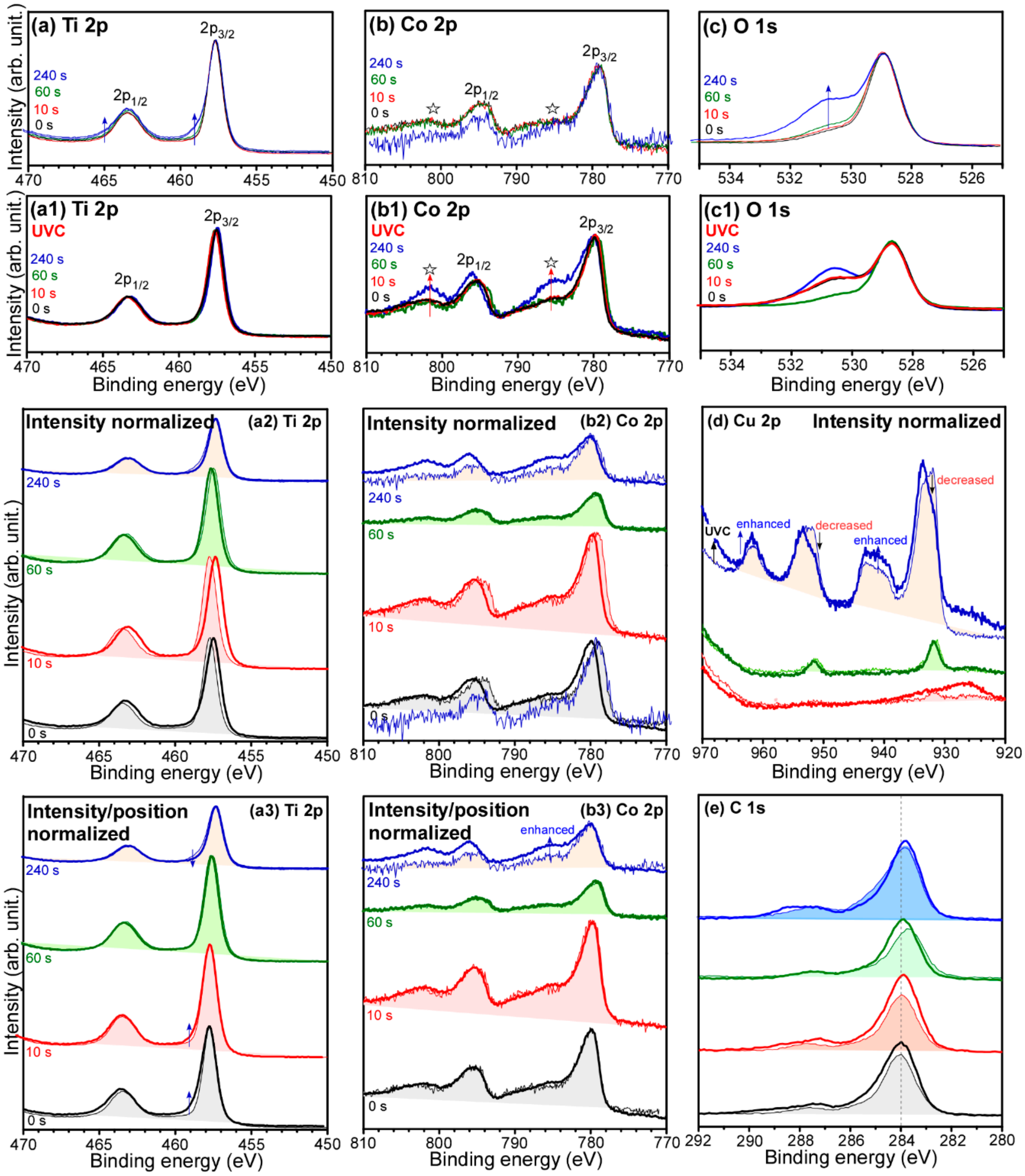

Figure S1. Normalized Ti 2p (**a** and **a1**), Co 2p (**b** and **b1**) and O 1s (**c** and **c1**) XPS profiles with Cu deposition time before and after UV CO_2 reduction, respectively, intensity-normalized (**a2**, **b2** and d), and intensity (and peak position)-normalized (**a3** and **b3**) Ti 2p (**a2** and **a3**), Co 2p (**b2** and **b3**), and Cu 2p (**d**) XPS profiles with Cu deposition time before and after UV CO_2 reduction, respectively, and C 1s XPS profiles (**e**) with Cu deposition time before and after UV CO_2 reduction.

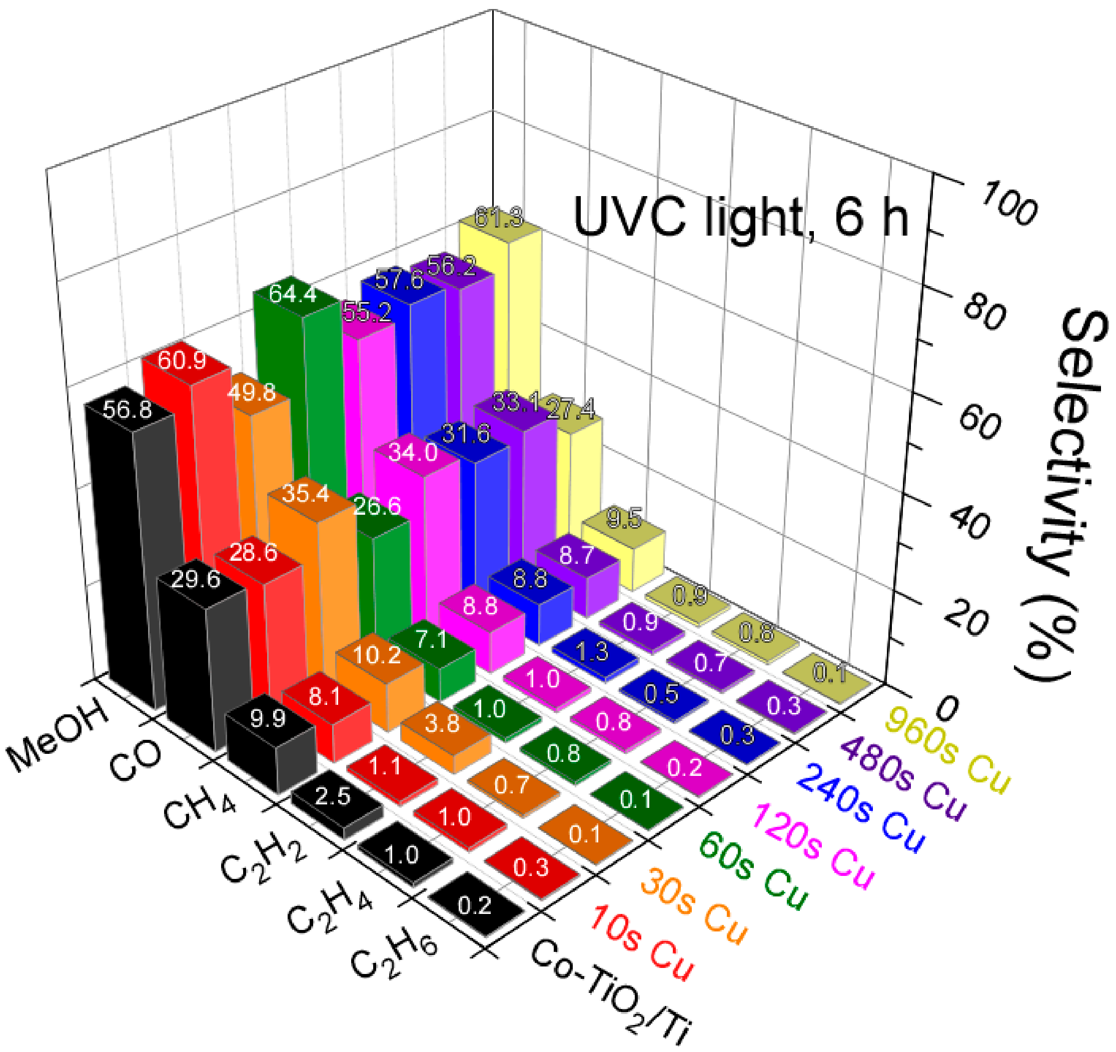

Figure S2. Photocatalytic CO_2 reduction selectivities.

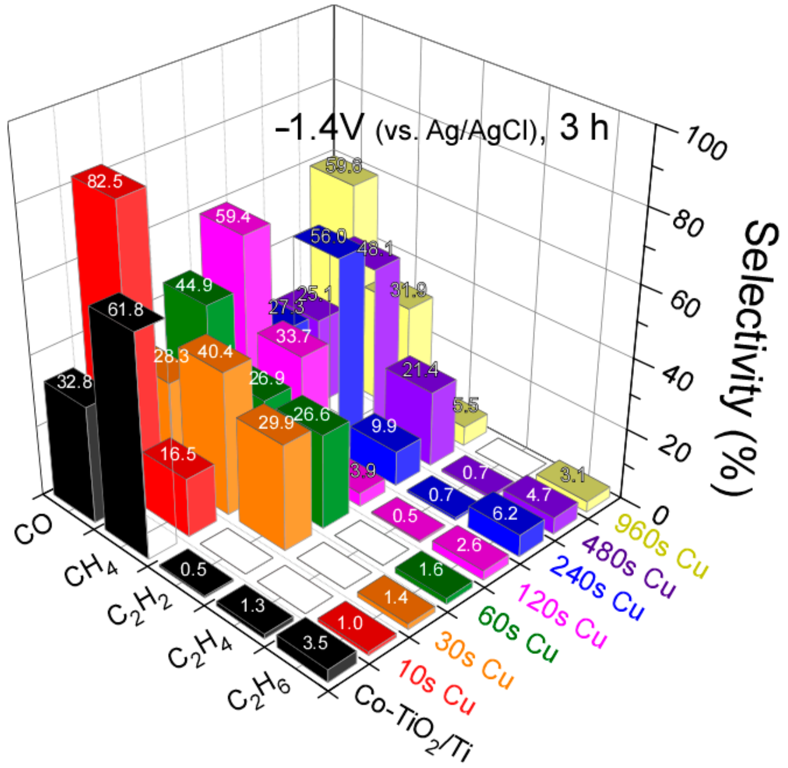

Figure S3. Electrocatalytic CO_2 reduction selectivities only with the C_n compounds. The total production selectivity of C_n compounds was less than 1% when compared with H_2. All the catalysts showed a H_2 production selectivity of >99%.

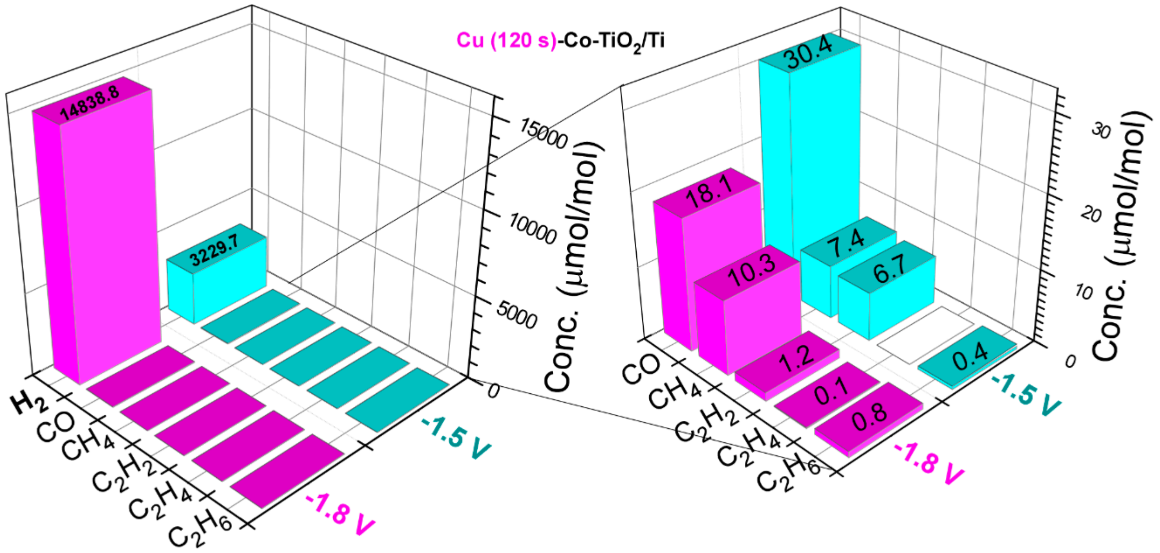

Figure S4. Electrochemical CO_2 reduction product yields at −1.5 V and −1.8 V for Cu (120 s)-Co-TiO_2/Ti catalysts. The right graph is the rescaled version for the products with low yields.
